# Standardised versus individualised multiherb Chinese herbal medicine for oligomenorrhoea and amenorrhoea in polycystic ovary syndrome: a randomised feasibility and pilot study in the UK

**DOI:** 10.1136/bmjopen-2016-011709

**Published:** 2017-02-03

**Authors:** Lily Lai, Andrew Flower, Philip Prescott, Trevor Wing, Michael Moore, George Lewith

**Affiliations:** 1 University of Southampton Primary Care and Population Sciences Aldermoor Health Centre Aldermoor Close, Southampton, UK; 2 University of Southampton, Mathematical Sciences Highfield Southampton, Southampton, UK; 3 Bournemouth University Centre for Ultrasound Studies, Bournemouth, UK

**Keywords:** Polycystic Ovary Syndrome, Chinese herbal medicine, Traditional Chinese Medicine, COMPLEMENTARY MEDICINE, PRIMARY CARE

## Abstract

**Objectives:**

To explore feasibility of a randomised study using standardised or individualised multiherb Chinese herbal medicine (CHM) for oligomenorrhoea and amenorrhoea in women with polycystic ovary syndrome (PCOS), to pilot study methods and to obtain clinical data to support sample size calculations.

**Design:**

Prospective, pragmatic, randomised feasibility and pilot study with participant and practitioner blinding.

**Setting:**

2 private herbal practices in the UK.

**Participants:**

40 women diagnosed with PCOS and oligomenorrhoea or amenorrhoea following Rotterdam criteria.

**Intervention:**

6 months of either standardised CHM or individualised CHM, 16 g daily taken orally as a tea.

**Main outcome measures:**

Our primary objective was to determine whether oligomenorrhoea and amenorrhoea were appropriate as the primary outcome measures for the main study. Estimates of treatment effects were obtained for menstrual rate, body mass index (BMI), weight and hirsutism. Data were collected regarding safety, feasibility and acceptability.

**Results:**

Of the 40 participants recruited, 29 (72.5%) completed the study. The most frequently cited symptoms of concern were hirsutism, weight and menstrual irregularity. Statistically significant improvements in menstrual rates were found at 6 months within group for both standardised CHM (mean difference (MD) 0.18±0.06, 95% CI 0.06 to 0.29; p=0.0027) and individualised CHM (MD 0.27±0.06, 95% CI 0.15 to 0.39; p<0.001), though not between group (p=0.26). No improvements were observed for BMI nor for weight in either group. Improvements in hirsutism scores found within group for both groups were not statistically significant between group (p=0.09). Liver and kidney function and adverse events data were largely normal. Participant feedback suggests changing to tablet administration could facilitate adherence.

**Conclusions:**

A CHM randomised controlled trial for PCOS is feasible and preliminary data suggest that both individualised and standardised multiherb CHMs have similar safety profiles and clinical effects on promoting menstrual regularity. These data will inform the design of a study in primary care that will incorporate an appropriate control.

**Trial registration number:**

ISRCTN 31072075; Results.

**Strengths and limitations of this study**

- This is the first study to evaluate multiherb Chinese herbal medicines in the UK for oligomenorrhoea and amenorrhoea in polycystic ovary syndrome.
- We compared standardised and individualised Chinese herbal medicines which were administered for 6 months.
- We prospectively collected data on safety and adverse events, menstrual regularity, hirsutism, anthropometrics and quality of life.
- We established feasibility of study procedures including recruitment, pharmacy randomisation, questionnaire administration, practitioner blinding and collecting adherence data.
- This feasibility study does not include a control group but which will be incorporated in a definitive trial in primary care.

## Introduction

Polycystic ovary syndrome (PCOS) affects an estimated 6–18% of women of reproductive age and is a heterogeneous condition characterised by endocrine and metabolic disturbances.^1–3^ Menstrual disturbances are common in PCOS, with oligomenorrhoea found in 47% of adults with PCOS and amenorrhoea in 19.2%.^4^ ^5^ Although the pathogenesis of PCOS is complex, it is typically associated with hyperandrogenism, hyperinsulinaemia and an elevated ratio of the gonadotropins luteinising hormone to follicle-stimulating hormone.^3^ Primary care management of menstrual disturbances typically involves oral contraceptives and insulin-sensitising agents but there is evidence of patient dissatisfaction associated with intolerable side effects, consequent poor adherence and potential increase in cardiovascular and metabolic risk.^6–10^ This highlights issues and barriers with current management and warrants exploration of other treatments.

Complementary medicines such as multiherb Chinese herbal medicines (CHMs) are reportedly used by over 70% of women with PCOS and is a commonly encountered condition among herbal practitioners.^11^ ^12^ Previous studies have demonstrated the potential for certain herbs for PCOS through mechanisms such as increasing granulosa production of oestradiol and progesterone, increasing aromatisation of testosterone to 17-β oestradiol and reducing levels of luteinising hormone.^13–15^ There is also emerging evidence from randomised controlled trials (RCTs) conducted in China and a pilot study conducted recently in the USA highlights the potential for CHM in the management of symptoms such as oligomenorrhoea and amenorrhoea.^16–20^ Although this provides encouraging preliminary evidence, the majority of the RCTs have been conducted in China and are methodologically poor, limiting the generalisability of these findings. This necessitates further exploration of CHM for PCOS within a fully powered RCT in UK primary care.

Given the novelty of offering CHM in UK primary care, it is important to first conduct a feasibility study to reduce uncertainties and facilitate planning of a main study.^21–24^ The aims of this study were to evaluate the feasibility of a randomised study exploring the effects of standardised or individualised multiherb CHM for PCOS-related oligomenorrhoea and amenorrhoea, to pilot study processes and to obtain safety and clinical data to facilitate sample size calculations for a main study.

## Materials and methods

### Design

#### Design and setting

Details of the study design are provided in our protocol publication.^25^ To summarise our methods, our primary feasibility research question was:

1. Is oligomenorrhoea and amenorrhoea appropriate as the primary outcome measure for the main study?

Our secondary feasibility questions were:

2. Are other measures more appropriate for investigation as the primary outcome measure for the main study?
3. What is the safety profile of CHM?
4. How should the CHM intervention in the main study be delivered?
5. Can a double-blind, randomised trial with CHM for PCOS be conducted?

This was a prospective, multicentre, pragmatic study in private CHM practices in the UK. Participants were randomised to one of two parallel arms comparing standardised multiherb CHM treatment against individualised multiherb CHM treatment. Although individualised CHM is regarded as ‘gold standard’ by the clinical literature^26^ and by an earlier Delphi study we conducted,^27^ this has limited application in UK primary care. The comparison between standardised and individualised CHM is therefore important to inform the final trial design and to maximise model validity for CHM practice.

Recruitment took place in the community inviting self-referrals by displaying ethics-approved posters and posts in community pharmacies, community noticeboards, online forums, press advertising and social media. Participants were included if they^1^ were women aged 18–44,^2^ presented with oligomenorrhoea or amenorrhoea and^3^ received a diagnosis of PCOS consistent with Rotterdam criteria.^28^ Women were excluded if they^1^ presented with other causes of hyperandrogenism or menstrual irregularities,^2^ were currently or suspected to be pregnant, or actively trying to conceive,^3^ had been breast feeding in the past 6 months,^4^ were receiving prohibited treatments such as hormonal contraceptives,^5^ had a history of liver or kidney pathologies,^6^ had a history of psychotic illness or eating disorders,^7^ had currently active major depression,^8^ were at risk of harmful and hazardous drinking,^9^ reported known allergies to herbal ingredients within standardised CHM,^10^ did not possess spoken or written language skills necessary to participate,^11^ were unable to attend proposed study visits,^12^ presented with abnormal liver and/or kidney function at screening. Diagnosis of PCOS was confirmed through participants providing medical letters and reports from previous medical investigations. Where a diagnosis remained unclear, participants were offered further blood tests and ultrasonography at a private clinic to confirm eligibility. On randomisation and with permission from each participant, their primary care provider was contacted in writing notifying them of their patient's study participation and of the results of any further investigations carried out as part of eligibility assessment.

We required a sample size of 40, based on requiring data from 15 participants per arm to calculate estimates of treatment effect and associated variability to inform the power calculation for a main study. We accounted for a 25% dropout rate based on similar studies.^29^ ^30^

Both CHM treatments were dispensed by Phoenix Medical UK and which supplied granulated extracts manufactured in China by Jiangyin Tianjiang Pharmaceutical Company Limited, a certified good manufacturing practice company. Participants were prescribed a dose of 16 g of granulated extracts per day for 6 months and were asked to prepare the granulated extracts twice a day by reconstituting 8 g of granulated extracts each time with hot water and taking it as a tea. This dose and length of treatment had been informed by our Delphi study.^27^ Our standardised CHM prescription was developed by this research team and was informed by common CHM treatment strategies highlighted by practitioners in our Delphi study.^27^ This prescription was standardised to contain 14 CHMs commonly used in PCOS, the contents of which can be found in table 1. In Chinese medicine terms based on traditional use, this prescription is directed at moving stagnant liver Qi, tonifying the kidney yang and on nourishing and moving the blood. It is not licensed as a proprietary product in the UK but, like individualised prescriptions, is available following a one-to-one consultation with a CHM practitioner in the UK. CHM practitioners in the UK must show evidence of an adequate level of education and training from an accredited institution, or equivalent experience, in order to be registered with a professional organisation such as the Register of Chinese Herbal Medicine (RCHM) or Association of Traditional Chinese Medicine (ATCM). Individualised CHM prescriptions permitted practitioners to prescribe treatment as usual from a range of 270 individual CHMs, and the details of the 20 most commonly prescribed herbs in the individualised CHM group have been provided as an online supplementary data file. No further training regarding individualised CHM prescribing was provided by the study team. Details regarding qualitative testing and characteristics of the granulated extracts have been provided previously.^25^

**Table 1:** Contents of standardised Chinese herbal medicine prescription

Chinese Pinyin name	Common name	Family name	Part used	Botanical name	Dried herbal daily dosage (g)	Percentage
Bai Shao (Chao)	Peony (dry fried)	Paeoniaceae	Root	*Paeonia lactiflora* Pall.	15	10.64
Chai Hu	Bupleurum	Umbelliferae	Root	*Bupleurum chinense* DC.	9	6.38
Chen Pi	Tangerine peel	Rutaceae	Peel	*Citrus reticulatata* Blanco	9	6.38
Chuan Xiong	Szechwan lovage rhizome	Umbelliferae	Rhizome	*Ligusticum chuanxiong* Hort.	9	6.38
Dang Gui Wei	Angelica extremitas	Umbelliferae	Root tail	*Angelica sinensis* (Oliv.) Diels	9	6.38
Gan Cao (Mi Zhi)	Liquorice root (honey-fried)	Fabaceae	Root	*Glycyrrhiza uralensis* Fisch.	6	4.26
Gou Qi Zi	Goji berry	Solanaceae	Fruit	*Lycium barbarum* L.	9	6.38
Gui Zhi	Cinnamon twig	Lauraceae	Twig	*Cinnamomum cassia* Presl.	9	6.38
Hong Hua	Safflower	Asteraceae	Flower	*Carthamus tinctorius* L.	9	6.38
Tao Ren	Peach kernel	Rosaceae	Seed	*Prunus persica* (L.) Batsch.	9	6.38
Tu Si Zi	Chinese dodder seed	Convolvulaceae	Seed	*Cuscuta chinensis* Lam.	12	8.52*
Xiang Fu (Cu Zhi)	Purple nutsedge (vinegar-fried)	Cyperaceae	Rhizome	*Cyperus rotundus* L.	12	8.52*
Yi Mu Cao	Motherwort	Lamiaceae	Top	*Leonurus japonicus* Houtt.	15	10.64
Zhi Ke	Bitter orange	Rutaceae	Mature fruit	*Citrus aurantium* L.	9	6.38
			Total prescription weight		141	

*Rounded up for column total to equal 100%.

10.1136/bmjopen-2016-011709.supp1 supplementary data

CHM treatment was offered for 6 months by a practitioner who needed to^1^ have a minimum 5 years in practice,^2^ be registered with a professional CHM organisation in the UK,^3^ be fully insured,^4^ have access to liver and kidney function testing, and^5^ agree to conduct procedures in the protocol. Dietary and lifestyle advice was permitted as part of usual care. Participants were blinded in that they were informed they would be randomised to one of two CHM treatments and were provided no further information regarding treatment differences. An independent statistician (PP) used computer-generated random numbers with allocation ratio 1:1 to provide an irregular block allocation sequence. Allocation codes were transferred to sealed opaque envelopes and provided to an RCHM-approved herbal dispensary who conducted randomisation. As practitioners were providing care as well as conducting objective outcome assessments, it was especially important to this study that practitioner and assessor blinding was maintained. In order to carry this out, practitioners were asked to formulate an individualised prescription for each participant at each visit. This was sent to the herbal dispensary who conducted randomisation at first visit, and dispensed either the standardised or individualised prescription according to randomisation. Prior to and during the study, practitioners and the study team did not have knowledge of the randomisation sequence or of treatment allocation. Participant blinding was not evaluated since participants were not informed regarding the differences between the two CHM treatments. Statistical analysis was conducted blinded.

Our feasibility outcomes were recruitment and retention rates. We included adherence as a part of our feasibility assessment, measured by Morisky Medication Adherence Scale (MMAS) at weeks 4 and at final visit, and by weighing prescription containers at week 12 and at final visit. Practitioner blinding was evaluated through a questionnaire used in previous studies of similar nature and analysed using Bang Blinding Index (BBI) administered at weeks 4, 12 and at final visit.^31^ We evaluated participant feedback at final visit and case report form (CRF) data were collected throughout the study as part of usual research practice and analysed to explore areas of potential improvement. At end of study, we conducted an audit of randomisation and allocation procedures to assess security of these processes.

Our primary outcome measure was menstrual regularity; secondary measures were hirsutism using validated modified Ferriman-Gallwey (mFG) questionnaire, anthropometrics using body mass index (BMI), weight, waist circumference, waist-to-hip ratio (WHR) and quality of life questionnaires Polycystic Ovary Syndrome Questionnaire (PCOSQ), Measure Yourself Medical Outcome Profile (MYMOP) and Dermatology Life Quality Index (DLQI). Additional information regarding the specific measures we used and clinical relevance of the individual scales and scores used are available in our study protocol.^25^ Although menstrual regulation is regarded as clinically important, we wished to explore menstrual regulation as the primary outcome for the main study because we remained uncertain regarding the effects of a 6-month CHM treatment on menstruation. We therefore collected clinical data on menstruation to obtain an estimate of treatment effect and associated variability to support sample size calculations for a future study. The appropriateness of menstrual regulation as a primary outcome for the main study was examined by seeing whether or not we could collect menstrual data for analysis. Furthermore, we explored whether menstrual regulation appeared to be an important outcome to participants by ranking patient-generated MYMOP data whereby participants specified up to two key symptoms (MYMOP1 and optionally MYMOP2) that were of greatest concern to them and which did not have to be PCOS-related. To investigate whether other outcome measures such as mFG or anthropometrics could be more appropriate as the primary outcome for the main study, we again collected data on treatment effects and where these symptoms ranked in MYMOP to indicate importance to participants.

Outcome measures were taken at baseline and at final visit with the exception of menstrual frequency which was recorded throughout study participation. Objective measures were assessed by the practitioner, and subjective measures were completed by participants in the absence of the practitioner. Liver and kidney function were assessed using alanine aminotransferase (ALT) and creatinine at baseline, week 4 and at final visit.

Our statistical analysis has been published previously in our study protocol.^25^ We planned to pilot statistical analysis in this feasibility study. Continuous variable comparisons were assessed at end of study adjusted for baseline assessments for the two groups using analysis of variance, or an analysis of covariance (ANCOVA) to include factors such as demographic variables. Categorical data were analysed using χ^2^ tests. Where assumptions of normality were not met, non-parametric methods were used. Since no formal power calculations have been carried out, results from the statistical analysis should be considered preliminary. Textual data collected from sources such as clinical notes and feedback questionnaires were analysed using thematic analysis.

This trial was registered with Current Controlled Trials (ISRCTN 31072075) prior to recruitment.

## Results

### Recruitment and study participation

Three practitioners responded to our request for volunteer for this study, but were unable to assist due to lacking access to safety testing (n=1) and lacking time (n=2). One practitioner, the main author (LL), provided all treatment from two sites in London and Hertfordshire.

We recruited 40 participants from 245 enquiries (16.3% eligibility rate) between January 2013 and July 2013; 28 (70%) in London, and 12 (30%) in Hertfordshire. The last participant visit was in February 2014. The most common reasons for ineligibility were receiving prohibited treatment (n=34), having regular periods (n=27) and distance to sites (n=22). Treatment groups were comparable at baseline for demographics (table 2) and outcome measures. Previous use of conventional medication was high in both groups (standardised group n=16, 80%; individualised group n=11, 55%), as was previous use of complementary and alternative medicine (CAM; standardised group n=14, 70%; individualised group n=16, 80%) and previous use of acupuncture or CHM (standardised group n=8, 40%; individualised group n=12, 60%).

**Table 2:** Baseline demographics for participants

Baseline demographics, n (%) unless specified otherwise	Standardised (n=20)	Individualised (n=20)
Age, mean (SD)	28.5 (6.0)	30.4 (6.6)
Years since diagnosis, mean (SD)	7.1 (4.8)	9.3 (6.7)
Menstrual presentation		
Oligomenorrhoea	15 (75)	17 (85)
Amenorrhoea	5 (25)	3 (15)
Current conventional medication use		
Yes	1 (5)	2 (10)
No	19 (95)	18 (90)
Of which previously used conventional medication*	16 (80)	11 (55)
Prior use of		
CAM: yes	14 (70)	16 (80)
Acupuncture or CHM: yes	8 (40)	12 (60)
Parity ≥1		
Yes	4 (20)	1 (5)
No	16 (80)	19 (95)
Ethnicity		
White British	7 (35)	9 (45)
White: Irish and other white	3 (15)	6 (30)
Mixed	3 (15)	1 (5)
Asian: Indian, Pakistani, Bangladeshi and other Asian	2 (10)	0
Black Caribbean and Black African	2 (10)	2 (10)
Chinese	1 (5)	0
Other/prefer not to answer	2 (10)	2 (10)
PCOS phenotype*		
Type A: menstrual disturbances, hyperandrogenism and polycystic ovaries	15 (75)	10 (50)
Type B: menstrual disturbances, hyperandrogenism	0	1 (5)
Type D: menstrual disturbances and polycystic ovaries	5 (25)	9 (45)
Employment status*		
Full-time employment	12 (60)	11 (55)
Part-time employment	2 (10)	3 (15)
Self-employed	2 (10)	2 (10)
Student	1 (5)	3 (15)
Homemaker	1 (5)	1 (5)
Out of work, looking for work	2 (10)	0

*Not compared at baseline.

CAM, complementary and alternative medicine, defined as prior use of one or more CAM treatments including Chinese herbal medicine, acupuncture, Western herbal medicine, nutritional supplements, homeopathy, aromatherapy, reiki healing, hypnotherapy, osteopathy, chiropractic or other (request to specify).

CHM, Chinese herbal medicine.

**Figure 1:**
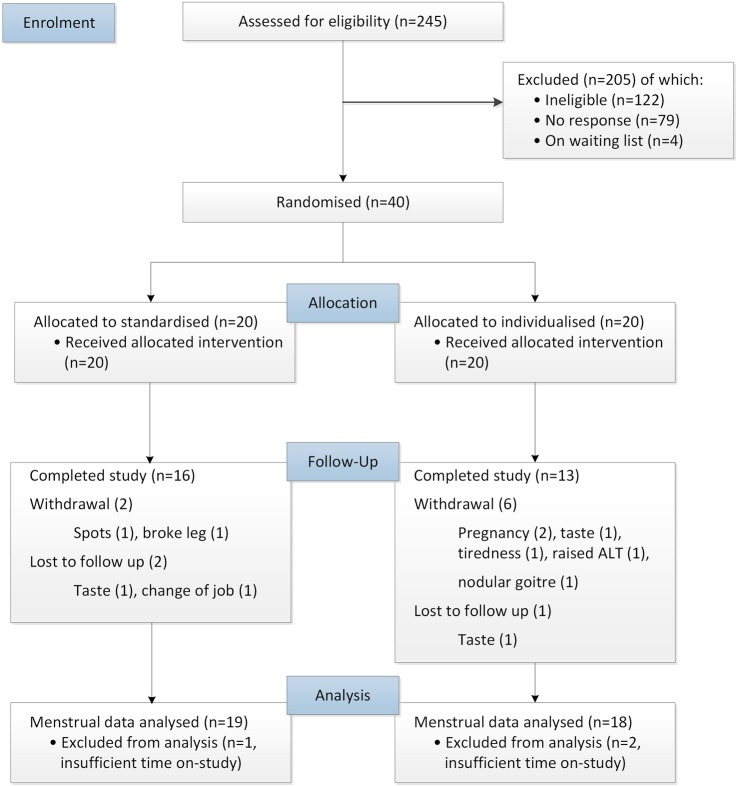
CONSORT participant flow chart. ALT, alanine aminotransferase.

Our participant flow diagram (figure 1) shows that 29 participants (72.5%) completed the 6-month treatment, 8 (20%) withdrew and 3 (7.5%) were lost to follow-up. Details of withdrawals and loss to follow-up are provided in later sections.

### Clinical data

#### Primary outcome: menstrual regularity at 6 months

Insufficient menstrual data at baseline and at end of study meant it was not possible to compare mean and variability of cycle lengths as our predefined assessment of menstrual regularity. We subsequently followed methods published in other PCOS studies by calculating a menstrual rate per 28 days and by dichotomising to responders and non-responders.^32^ ^33^ Clinically, this means that normal menstrual rate of 9–12 menstrual cycles per year equates to a menstrual rate of 0.75–1 per month, oligomenorrhoea defined as 3–8 menstrual cycles per year equates to 0.25–0.67 per month, and amenorrhoea as being <0.25 per month.

We analysed menstrual data from 19 participants on standardised CHM and 18 participants on individualised CHM. Data were unavailable from three participants who withdrew or were lost to follow-up within 21 days postrandomisation.

ANCOVA of mean menstrual rate at 6 months, adjusting for baseline menstrual rate and age, was not statistically significant between group (mean difference (MD) 0.10±0.08, 95% CI −0.07 to 0.26; p=0.26). Statistically significant changes in menstrual rates were seen within group for both standardised CHM (MD 0.18±0.06, 95% CI 0.06 to 0.29; p=0.0027) and individualised CHM (MD 0.27±SE 0.06, 95% CI 0.15 to 0.39; p<0.001; table 3).

**Table 3:** Menstrual rate at baseline and 6 months

	Menstrual rate (menses/28 days)					
	Standardised group		Individualised group		Between group	
Assessment	n	Mean±SE (95% CI)	n	Mean±SE (95% CI)	Adjusted mean difference±SE (95% CI)	p Value
Baseline	20	0.38±0.06 (0.25 to 0.52)	20	0.51±0.06 (0.38 to 0.64)	NA	
6 months menstrual rate*	19	0.62±0.06 (0.50 to 0.73)	18	0.71±0.06 (0.60 to 0.83)	0.10±0.08 (−0.07 to 0.26)	0.26
Change from baseline at 6 months*	19	0.18±0.06 (0.06 to 0.29)	18	0.27±0.06 (0.15 to 0.39)		

*Adjusted for covariates baseline menstrual rate (p=0.01) and age (p=0.03).

NA, not available.

We dichotomised menstrual data into responders and non-responders by defining response as a change from amenorrhoea to either oligomenorrhoea or eumenorrhoea, a change from oligomenorrhoea to eumenorrhoea, or as achieving pregnancy. Comparing response rates between standardised group (n=10 of 19, 52.6%) versus individualised group (n=13 of 18, 72.3%), this did not reach statistical significance between group (OR 1.37, 95% CI 0.82 to 2.29, p=0.22).

#### Secondary outcomes

ANCOVA of prespecified secondary measures, adjusting for baseline measures and age, were used to compare between-group differences (table 4). While our main analysis focuses on change scores, we have additionally provided baseline and postintervention scores, adjusted for age and baseline scores, in an online supplementary data file for interested readers.

**Table 4:** ANCOVA comparing secondary outcome measures within and between group

Outcome measure by group	n	Adjusted mean difference from baseline	SE	95% CI		p Value	p Value between group
				Lower limit	Upper limit		
Modified Ferriman-Gallwey score							
Standardised	15	−2.52	0.42	−3.40	−1.65	<0.001	0.09
Individualised	14	−1.44	0.44	−2.35	−0.54	0.001	
BMI (kg/m^2^)							
Standardised	15	−0.41	0.46	−1.40	0.54	0.37	0.83
Individualised	15	−0.26	0.48	−1.25	0.72	0.59	
Weight (kg)							
Standardised	15	−1.13	1.30	−3.80	1.54	0.38	0.85
Individualised	14	−0.77	1.34	−3.53	1.99	0.57	
Waist-to-hip ratio							
Standardised	15	0.002	0.01	−0.02	0.02	0.84	0.02*
Individualised	12	−0.04	0.01	−0.06	−0.01	<0.001	
Waist circumference (cm)							
Standardised	15	−0.80	1.28	−3.45	1.85	0.53	0.27
Individualised	14	−2.96	1.38	−5.81	−0.11	0.03	
MYMOP1							
Standardised	17	2.04†	0.39	1.24	2.84	<0.001	0.98
Individualised	18	2.02†	0.38	1.24	2.80	<0.001	
MYMOP2							
Standardised	17	0.87	0.39	0.07	1.67	0.03	0.29
Individualised	18	1.46	0.38	0.68	2.24	<0.001	
MYMOP activity							
Standardised	11	2.38†	0.43	1.49	3.26	<0.001	0.19
Individualised	15	1.59	0.36	0.84	2.34	<0.001	
MYMOP well-being							
Standardised	16	0.92	0.32	0.28	1.57	0.004	0.97
Individualised	18	0.90	0.30	0.30	1.51	0.003	
PCOSQ total							
Standardised	17	5.17	1.0	3.09	7.25	<0.001	0.33
Individualised	18	3.74	1.00	1.72	5.77	<0.001	
PCOSQ emotions							
Standardised	17	0.97†	0.22	0.53	1.42	<0.001	0.77
Individualised	18	1.06†	0.21	0.63	1.49	<0.001	
PCOSQ body hair							
Standardised	17	0.84	0.26	0.31	1.38	0.001	0.16
Individualised	18	0.32	0.26	−0.20	0.83	0.11	
PCOSQ weight							
Standardised	17	0.79	0.32	0.15	1.44	0.01	0.61
Individualised	18	0.56	0.31	−0.06	1.19	0.07	
PCOSQ infertility							
Standardised	17	1.17†	0.25	0.66	1.68	<0.001	0.26
Individualised	18	0.77	0.24	0.27	1.27	0.001	
PCOSQ menstrual							
Standardised	17	1.40†	0.24	0.91	1.88	<0.001	0.28
Individualised	18	1.03†	0.23	0.56	1.50	<0.001	
DLQI							
Standardised	16	−1.97	0.66	−3.32	−0.62	0.003	0.09
Individualised	18	−0.36	0.62	−1.63	0.91	0.56	

*Favouring individualised CHM.

†Achieved MCID.

BMI, body mass index; DLQI, Dermatology Life Quality Index; MCID, minimum clinically important difference; MYMOP, Measure Yourself Medical Outcome Profile; PCOSQ, Polycystic Ovary Syndrome Questionnaire.

10.1136/bmjopen-2016-011709.supp2 supplementary data

##### Menstrual regularity at end of study

Owing to final visit arrangements, participation extended beyond 6 months for 26 participants. The nature of menstrual reporting meant these data continued to be collected until their final visit and we conducted a post hoc analysis to explore the effects of this additional data on menstrual rate.

Median days on study for standardised CHM was 175 days (IQR 167–180) and for individualised CHM was 177.5 days (IQR 167–190). As in our primary analysis, between-group differences were not statistically significant (MD 0.10±0.09, 95% CI −0.09 to 0.29; p=0.27). At final visit, changes from baseline were again statistically significant for standardised CHM (MD 0.25±0.06, 95% CI 0.12 to 0.38; p<0.001) and individualised CHM (MD 0.35±0.07, 95% CI 0.22 to 0.49; p<0.001).

##### Hirsutism

Statistically significant reductions in mFG scores from baseline were seen in both standardised group (MD −2.52±0.42, 95% CI −3.40 to −1.65; p<0.001) and individualised group (MD −1.44±0.44, 95% CI −2.35 to −0.54; p=0.001) but which did not reach between-group significance (p=0.09). Patient-reported quality of life relating to hirsutism is presented as part of the PCOSQ data in Polycystic Ovary Syndrome Questionnaire section.

##### Anthropometrics

Changes observed in BMI and weight were not statistically significant within or between group. Statistically significant changes in waist circumference were apparent within group for individualised CHM (MD −2.96±1.38, 95% CI −5.81 to −0.11; p=0.03) but were seen neither in standardised CHM (MD −0.8±1.28, 95% CI −3.45 to 1.85; p=0.53), nor between group (MD −2.16±1.90, 95% CI −6.08 to 1.75; p=0.27). Individualised CHM also saw statistically significant within-group changes from baseline in WHR; MD −0.04±0.01, 95% CI −0.06 to 0.01; p<0.001) but which were not seen in standardised CHM (MD 0.002±0.01, 95% CI −0.02 to 0.02; p=0.84). This difference was statistically significant between groups favouring individualised CHM (p=0.02).

##### Measure Yourself Medical Outcome Profile

Count and frequency ranking of MYMOP1 and MYMOP2 data indicated that the three most distressing concerns were hirsutism (n=19), weight (n=16) and menstrual regularity (n=12).

For both groups, statistically significant improvements were seen within group for all four MYMOP submeasures of MYMOP1 (standardised p<0.001; individualised p<0.001), MYMOP2 (standardised p=0.03; individualised p<0.001), MYMOP activity (standardised p<0.001; individualised p<0.001) and MYMOP well-being (standardised p=0.004; individualised p=0.003). There were however no between-group differences in any of these measures (MYMOP1 p=0.98, MYMOP2 p=0.29, MYMOP activity p=0.19, MYMOP well-being p=0.97). Minimum clinically important difference (MCID) of 1.0 was reached for MYMOP1 for both groups and for MYMOP activity for only standardised CHM. All remaining MYMOP results show potential for a clinically important difference since 95% CIs include the MCID.

##### Polycystic Ovary Syndrome Questionnaire

For both groups, statistically significant improvements were seen in PCOS total scores within group for both standardised CHM (MD 5.17±1.0, 95% CI 3.09 to 7.25; p<0.001) and individualised CHM (MD 3.74±1.0, 95% CI 1.72 to 5.77; p<0.001) but not between group (p=0.33). Statistically significant improvements were seen within group for all PCOSQ domains for standardised CHM for emotions (p<0.001), body hair (p=0.001), weight (p=0.01), infertility (p<0.001) and menstrual (p<0.001); and for individualised CHM for emotions (p<0.001), infertility (p=0.0010) and menstrual (p<0.001) domains only. No between-group differences were detected. The MCID of 0.5 was reached for emotions and menstrual domains for both groups, and for infertility in only the standardised group. All remaining PCOSQ domain scores show potential for a clinically important difference.

##### Dermatology Life Quality Index

A statistically significant within-group change in DLQI score was seen for standardised treatment (p=0.003) but not in individualised treatment (p=0.56) and which was not statistically significant between group (p=0.09). MCID of 3.2 was not met in either group but the 95% CI for standardised CHM includes 3.2, suggesting potential for clinical benefit.

### Safety and adverse events

#### Liver and kidney function

The vast majority of ALT and creatinine tests were normal for both treatments. However, at week 4, one participant in individualised CHM displayed normal creatinine but abnormal ALT readings and was withdrawn as a precautionary measure. This was later assessed as being alcohol related and not related to study medication.

#### Nature and duration of adverse events and adverse reactions

Adverse reactions (AR) were minor with nine reactions reported among six participants in standardised CHM and four participants in individualised CHM. All nine were assessed as mild in severity, expected, not serious and having a reasonable causal relationship with treatment. Gastrointestinal symptoms (bloating, nausea, loose stools, vomiting) were the most commonly reported with the remainder consisting of tiredness, skin breakout, ovulation pain, light headedness and headache.

The majority of AR incidents (14/17, 82.4%) subsided with continued administration and lasted a median of 4 days (range 1–74 days). Two symptoms (loose stools and bloating) persisted with continued administration of standardised CHM for two participants.

Two serious adverse events were reported. One participant allocated to individualised CHM was diagnosed with nodular goitre and one participant allocated to standardised CHM experienced a leg fracture. Causality for both events was assessed by hospital consultants independent of the study and confirmed to be unrelated to study treatment.

#### Pregnancy

Two participants taking individualised CHM became pregnant on study and were withdrawn for monitoring. Both reported healthy pregnancies and uncomplicated live births at full term.

### Process evaluation

#### Blinding

BBIs were calculated from practitioner blinding data at weeks 4 (n=36) and 12 (n=31) and final visit (n=36) following published methods.^31^ ^34^ At week 4, this was standardised (BBI −0.11, 95% CI −0.35 to 0.14, random) and individualised (BBI 0.47, 95% CI 0.23 to 0.71, unblinded); week 12 standardised (BBI −0.24, 95% CI −0.54 to 0.07, random) and individualised (BBI 0.50, 95% CI 0.12 to 0.88, unblinded); and final visit standardised (BBI −0.56, 95% CI −0.91 to −0.20, opposite) and individualised (BBI 0.61, 95% CI 0.30 to 0.92, unblinded). The most commonly cited reasons for treatment guesses were ‘presence of effects’ and ‘lack of effects’ which consistently led to guesses of individualised and standardised CHM, respectively.

#### Security of randomisation and allocation procedures

Randomisation, allocation and CHM dispatch were conducted as intended, after comparing the randomisation list from our statistician against the randomisation log generated by the pharmacy and a sample of 89 (39.9%) prescriptions.

#### Adherence

Both groups demonstrated a small increase in MMAS mean scores by the final visit (standardised MD 0.9±SD 2.2, 95% CI −0.3 to 2.0; individualised MD 1.0±1.4, 95% CI 0.3 to 1.8) which was not statistically significant between group (p=0.86). A mean of 65% (SD 21.2) of CHMs had been administered, calculated from 21 participants (52.5%) for whom at least 75% of prescription data had been accounted for.

#### Participant and CRF evaluation

Overall, participants reported positive experiences of the study. Thirty-two participants (80%) reported they would ‘definitely’ or ‘probably’ agree to take part in a study like this again. Three participants (7.5%) reported being ‘unsure’ or ‘probably not’. There were five non-responses (12.5%).

Thematic analysis of participant feedback highlighted five broad themes: (1) appraisal of the CHM intervention, (2) managing practicalities of research participation, (3) empowerment and enablement, (4) management of care and (5) quality of care. Participants appraised CHM by improvements in symptoms of personal concern and not necessarily menstrual regularity. Participants raised concerns regarding safety and taste difficulties that reduced adherence and described feeling empowered through gaining further knowledge of PCOS and of CHM within this study. Aspects of care management were highly valued such as frequent monitoring and a holistic and personalised approach. Quality of care was appraised positively and seen to be delivered in an approachable, non-judgemental and knowledgeable manner, and with compassion and trust.

To evaluate study processes, we analysed thematically CRF data which highlighted three broad themes: (1) improving participant experience, (2) improving trial conduct and management, and (3) design considerations for future research. Highlighted areas for improvement suggested improved consideration for participant anxieties surrounding taking anthropometric and hirsutism assessments. Management of CHMs was considered complex, particularly surrounding CHM administration and weighing which could have been discussed in more detail with participants. Participants reported wishing to finding out personal and overall study progress and recommended the inclusion of biochemical investigations and ovarian status in future research. Limitations with hirsutism assessment were raised as it did neither consider rate of hair growth nor area of hair distribution. Future work could consider frequency of hair removal or time until hair regrowth which may reflect more accurately hirsutism status as appraised by participants.

## Discussion

The findings of this study suggest that it is feasible to conduct a clinical study in the UK using an RCHM-approved dispensary, offering either individualised or standardised multiherb CHM treatment for 6 months to women with PCOS-related oligomenorrhoea and amenorrhoea. Our results support the evaluation of oligomenorrhoea and amenorrhoea, but suggest that hirsutism could be considered as a primary outcome since this was a key concern for our participants according to our MYMOP1 and MYMOP2 ranking data. Individualised and standardised CHMs when prescribed by a registered CHM practitioner and administered for 6 months appear to be safe, without harmful effects on either liver or kidney function, and with minimal side effects. Although these findings appear encouraging, they should be considered preliminary owing to the sample size and the feasibility nature of this study and our results relating to clinical effects and safety should be interpreted with these limitations in mind.

Our preliminary data support the use of either individualised or standardised CHM as there was no statistically significant difference between group in improvement of menstrual rate at 6 months. However, we acknowledge that this feasibility study had a small sample size and we cannot rule out the possibility that a larger sample would provide greater power to detect a potentially important difference. Although we considered the retention rate of 72.5% acceptable, changing administration to once per day and to a tablet or capsule are likely to further improve participant retention. Finally, we were unsuccessful in recruiting CHM practitioners and would recommend that a future study provide financial reimbursement for practitioner time and to plan for better access to safety testing.

For this study, we were unable to include a control arm such as placebo, wait list or treatment as usual and our results should be interpreted with caution owing to factors that were not controlled for such as contextual effects and regression to the mean. We however successfully evaluated feasibility as planned and a significant strength of this study is that we collected data prospectively on standardised and individualised multiherb CHMs which were administered according to real-world practice and for 6 months. Safety and adverse events data from our study appear to be consistent with the findings of previous CHM studies in the UK^29^ ^35^ ^36^ which suggest that CHMs appear to be safe and overall well tolerated. Our study compared real-world CHM-prescribing approaches for a significant duration of time, whereas the majority of previous PCOS and CHM trials evaluate standardised CHMs for <6 months.^18^ ^37^ Although our safety data are encouraging and appears consistent with the findings from an Australian survey,^38^ this was a small-scale study and larger studies with longer term follow-up remain warranted. Owing to financial constraints, we were unable to conduct biochemical tests and evaluate polycystic ovarian morphology as part of our outcome measures and we would strongly recommend including these in a main study to increase the rigour of the study design and to maximise relevance of such findings for clinicians and for the PCOS population. Future feasibility studies should consider including these PCOS-relevant medical investigations to increase scientific rigour.

We successfully recruited the target sample size and found an acceptable retention rate of 72.5%. This suggests strong interest from the PCOS population in the UK which is important since less than one-third of primary care trials are able to recruit within the specified timeframe^39^ and high attrition rates of up to 53% are typically found in PCOS studies.^40^ ^41^ However, we recruited outside of primary care and participants were aware they would be randomised to one of two active CHM treatments. A question that remains unanswered is the recruitment and retention rate within a National Health Service (NHS) setting and the willingness of participants to be randomised to a placebo or wait-list control group. Furthermore, it is possible that we recruited a selective group since a significant proportion of our participants reported previously discontinuing conventional medicines due to side effects or lack of effects, and had prior use of CAM and of Chinese medicine. However, we argue that this could potentially reflect real-world prescribing strategies where women resistant to first-line treatments such as the contraceptive pill or insulin-sensitising agents could instead be offered as therapeutic alternatives such as CHMs, provided there was robust evidence for clinical effect and safety.

Although evaluating oligomenorrhoea and amenorrhoea would be an appropriate primary outcome measure for the main trial, this feasibility study has highlighted challenges in statistical and clinical interpretation of menstrual regularity. Insufficient menstrual data at baseline and at end of study meant it was not possible to use our predefined approach of assessing menstrual regularity and this was thus evaluated as a menstrual rate following other PCOS studies.^25^ ^32^ ^33^ Although this enables our results to be more easily compared with the wider literature, we acknowledge this was a decision made post hoc. Furthermore, it is possible that our baseline menstrual diary data were subject to recall bias and which could be overcome by considering a run-in phase. As hirsutism ranked higher than menstrual irregularity as a key concern for participants in the MYMOP data, it is possible that evaluating hirsutism would be more relevant to patients with PCOS and which in turn could facilitate recruitment in a future study. This needs to be considered alongside shortcomings of hirsutism assessment we have already highlighted, suggesting that a subjective or quality of life measure may be more appropriate.

Robust evaluation of adherence and practitioner blinding is important in RCTs as a means of assessing internal validity. We found the process of collecting both self-reported and objective adherence data useful to triangulate findings and would recommend this for future trials. These data enabled us to conclude that a reduction in prescribed CHM dosage may be appropriate to improve participant acceptability and without compromising clinical effect on menstrual cyclicity. By close of study, our practitioner blinding results point to a ‘standardised opposite and individualised unblinded’ scenario, suggesting that blinding was likely secure. This highlights a ‘wishful thinking’ scenario that is consistent with other studies and suggests a high response bias, where the desire to believe that the more effective intervention—in this case the individualised treatment—was associated with a treatment response.^31^ ^42^ This emphasises the importance of blinding caregivers and assessors in RCTs and in evaluating blinding as a key factor in internal validity.

## Conclusions

We have demonstrated that it is feasible to conduct a CHM trial for oligomenorrhoea and amenorrhoea in PCOS. Our preliminary findings suggest that individualised and standardised multiherb CHMs have similar safety profiles and clinical effects on menstrual regularity. This evaluation supports the use of menstrual regularity as a primary outcome measure for a main study which should include an appropriate control group such as placebo or treatment as usual.
