# Supplementary material for: Standardised versus individualised multiherb Chinese herbal medicine for oligomenorrhoea and amenorrhoea in polycystic ovary syndrome: a randomised feasibility and pilot study in the UK
Source: BMJ Open. 2017 Feb 3;7(2):e011709. doi: 10.1136/bmjopen-2016-011709 (PMC5293993; doi:10.1136/bmjopen-2016-011709)
Supplement: supplementary data [file bmjopen-2016-011709supp1.pdf]

List of 20 most frequently prescribed Chinese herbs for individualised group in ORCHID study

	Chinese Pinyin Name	Product Name from Manufacturer Certificate of Analysis*	Frequency of Total Count (n=1533)	Percentage of Total Count
1	Chuan Xiong	Rhizoma Chuanxiong	77	5.02%
2	Xiang Fu(Cu Zhi)	Rhizoma Cyperi Prep.	77	5.02%
3	Chai Hu	Radix Bupleuri	69	4.50%
4	Bai Shao	Radix Paeoniae Alba	67	4.37%
5	Gan Cao(Mi Zhi)	Radix Et Rhizoma Glycyrrhizae Praeparata Cum Melle	61	3.98%
6	Bai Hua She She Cao	Herba Hedyotis Diffusae	55	3.59%
7	Dang Gui	Radix Angelicae Sinensis	49	3.20%
8	Shu Di Huang	Radix Rehmanniae Prep.	47	3.07%
9	Mu Dan Pi	Cortex Moutan	46	3.00%
10	Chi Shao(Chao)	Radix Paeoniae Rubra Prep.	45	2.94%
11	Gan Cao	Radix Et Rhizoma Glycyrrhizae	45	2.94%
12	Sheng Di Huang	Radix Rehmanniae	45	2.94%
13	Gui Zhi	Ramulus Cinnamomi	45	2.94%
14	Zhi Qiao / Zhi Ke(Fu Chao)	Fructus Aurantii Prep.	40	2.61%
15	Fu Ling	Poria	37	2.41%
16	Chen Pi	Pericarpium Citri Reticulatae	37	2.41%
17	Yan Hu Suo(Cu Zhi)	Rhizoma Corydalis Prep.	34	2.22%
18	Dang Gui Wei	Radix (Branch) Angelicae Sinensis	31	2.02%
19	Huang Qin	Radix Scutellariae	27	1.76%
20	Zhi Zi	Fructus Gardeniae	27	1.76%

*Manufacturer: TianJiang Pharmaceutical Company Ltd.
