# Supplementary material for: Standardised versus individualised multiherb Chinese herbal medicine for oligomenorrhoea and amenorrhoea in polycystic ovary syndrome: a randomised feasibility and pilot study in the UK
Source: BMJ Open. 2017 Feb 3;7(2):e011709. doi: 10.1136/bmjopen-2016-011709 (PMC5293993; doi:10.1136/bmjopen-2016-011709)
Supplement: supplementary data [file bmjopen-2016-011709supp2.pdf]

Baseline and adjusted post-intervention values for standardised and individualised Chinese herbal medicine groups

**Baseline characteristic**	**n**	**Standardised Mean (SE)**	**n**	**Individualised Mean (SE)**
Menstrual rate, n per 28 days				
Baseline	20	0.38 (0.06)	20	0.51 (0.06)
6m	19	0.62 (0.06)	18	0.71 (0.06)
Modified Ferriman-Gallwey score				
Baseline	20	8.05 (1.35)	20	7.60 (1.46)
6m	15	5.65 (0.42)	14	6.73 (0.44)
BMI (kg/m^2)				
Baseline	20	27.23 (1.29)	20	28.09 (1.76)
6m	15	27.21 (0.46)	14	27.35 (0.48)
Weight (kg)				
Baseline	20	74.49 (3.82)	20	78.20 (5.30)
6m	15	74.70 (1.30)	14	75.06 (1.34)
Waist hip ratio				
Baseline*	20	0.82 (0.02)	19	0.82 (0.01)
6m	15	0.83 (0.01)	12	0.79 (0.01)
Waist circumference (cm)				
Baseline	20	87.37 (3.31)	20	90.27 (4.46)
6m	15	87.49 (1.28)	13	85.33 (1.38)
MYMOP Score 1				
Baseline	20	4.60 (0.23)	20	5.15 (0.20)
6m	17	2.85 (0.39)	18	2.87 (0.38)
MYMOP Score 2				
Baseline	20	4.35 (0.22)	20	4.45 (0.28)
6m	17	3.62 (0.39)	18	3.03 (0.38)
MYMOP Activity				
Baseline[#]	14	3.93 (0.27)	16	4.81 (0.31)
6m	11	2.01 (0.43)	15	2.79 (0.36)
MYMOP Wellbeing				
Baseline[^]	19	3.37 (0.38)	20	3.20 (0.38)
6m	16	2.43 (0.32)	18	2.45 (0.30)
PCOSQ Total				
Baseline	20	18.25 (1.25)	20	17.28 (1.26)
6m	17	22.95 (1.02)	18	21.53 (0.99)
PCOSQ Emotions				
Baseline	20	3.91 (0.28)	20	3.36 (0.28)
6m	17	4.59 (0.22)	18	4.68 (0.21)
PCOSQ Body Hair				
Baseline	20	3.94 (0.43)	20	3.55 (0.48)
6m	17	4.43 (0.26)	18	3.90 (0.26)
PCOSQ Weight				
Baseline	20	3.62 (0.42)	20	3.22 (0.53)
6m	17	4.39 (0.32)	18	4.16 (0.31)
PCOSQ Infertility				
Baseline	20	3.39 (0.48)	20	3.59 (0.34)
6m	17	4.64 (0.25)	18	4.24 (0.24)

PCOSQ Menstrual				
Baseline	20	3.39 (0.21)	20	3.56 (0.25)
6m	17	4.91 (0.24)	18	4.54 (0.23)
DLQI				
Baseline	20	2.75 (0.75)	20	5.10 (1.48)
6m	16	2.15 (0.66)	18	3.76 (0.62)

Abbreviations: SE=Standard Error; BMI=Body Mass Index; MYMOP=Measure Yourself Medical Outcome Profile; PCOSQ=Polycystic Ovary Syndrome Questionnaire; DLQI=Dermatology Life Quality Index; *Measurement for one participant in individualised not possible since hip circumference exceeded limits of available equipment; #Participants could choose to specify an activity which they were impacted by, hence totals in each group do not equal 20; ^Missing data from one participant due to incomplete questionnaire; All post-intervention scores adjusted using age and baseline scores as co-variates
